# Polysaccharide‐based biomaterials in a journey from 3D to 4D printing

**DOI:** 10.1002/btm2.10503

**Published:** 2023-03-22

**Authors:** Hanieh Shokrani, Amirhossein Shokrani, Farzad Seidi, Mohammad Mashayekhi, Saptarshi Kar, Dragutin Nedeljkovic, Tairong Kuang, Mohammad Reza Saeb, Masoud Mozafari

**Affiliations:** ^1^ Jiangsu Co‐Innovation Center for Efficient Processing and Utilization of Forest Resources, International Innovation Center for Forest Chemicals and Materials Nanjing Forestry University Nanjing China; ^2^ Department of Chemical Engineering Sharif University of Technology Tehran Iran; ^3^ Department of Mechanical Engineering Sharif University of Technology Tehran Iran; ^4^ College of Engineering and Technology, American University of the Middle East Kuwait; ^5^ College of Material Science and Engineering, Zhejiang University of Technology Hangzhou China; ^6^ Department of Polymer Technology, Faculty of Chemistry Gdańsk University of Technology Gdańsk Poland; ^7^ Department of Tissue Engineering & Regenerative Medicine Iran University of Medical Sciences Tehran Iran

**Keywords:** 3D printing, 4D printing, bioprinting, carbohydrate polymers, polysaccharides, translational medicine

## Abstract

3D printing is a state‐of‐the‐art technology for the fabrication of biomaterials with myriad applications in translational medicine. After stimuli‐responsive properties were introduced to 3D printing (known as 4D printing), intelligent biomaterials with shape configuration time‐dependent character have been developed. Polysaccharides are biodegradable polymers sensitive to several physical, chemical, and biological stimuli, suited for 3D and 4D printing. On the other hand, engineering of mechanical strength and printability of polysaccharide‐based scaffolds along with their aneural, avascular, and poor metabolic characteristics need to be optimized varying printing parameters. Multiple disciplines such as biomedicine, chemistry, materials, and computer sciences should be integrated to achieve multipurpose printable biomaterials. In this work, 3D and 4D printing technologies are briefly compared, summarizing the literature on biomaterials engineering though printing techniques, and highlighting different challenges associated with 3D/4D printing, as well as the role of polysaccharides in the technological shift from 3D to 4D printing for translational medicine.

## 1 INTRODUCTION

Polysaccharides, mainly chitosan,^1^ alginate,^2^ agarose,^3^ starch,^4^ glycogen,^5^ and cellulose,^6^ as well as their blends^7^ have been widely used for biomedical purposes, ranging from imaging and diagnostic to therapeutic, delivery and biosensing applications.^8^, ^9^ The bioactivity of polysaccharides gives reason for their usage in the treatment of diseases such as antitumor, antivirus, and immunoregulatory.^10^, ^11^ Although polysaccharides are best known for appropriate biocompatibility and nontoxic nature, they suffer from poor mechanical properties. Therefore, there have been several kinds of research in which surface‐grafted^12^ and crosslinked^13^ polysaccharides have been employed for drug and gene delivery systems as well as electroconductive hydrogels.^14^, ^15^, ^16^ Another limitation of polysaccharides could be difficulty of purification and extraction. There are also some reports emphasizing that the stability of polysaccharide‐based scaffolds is limited in biological media, necessitating modification of polysaccharides and their extraction as well as processing circumstances.^17^ Thus, there was a need for novel technologies manufacturing predesigned polysaccharide‐based biomaterials, like electrospinning.^18^

3D printing or additive manufacturing (AM) enables one to print a series of materials in a layer‐by‐layer manner, with the potential to control the shape and properties of each layer. The resultant structure from a 3D printer is usually a complex, customized, and solid one already formed as an image in a digital brain. In a brief classification, AM can be categorized into five main groups, including inject printing, binder jetting, extrusion‐based printing, selective laser sintering (SLS), and stereolithography (SLA).^19^, ^20^ This modern technology enjoys several advantages in comparison with the classical processing methods. For instance, AM has a great capability of reproducibility, appropriate control over the fabrication process, individualization of product series and facile modification of the final products. In the field of biomedical engineering, the ability to fabricate different shapes (meniscus, bone, nose, and ear) with excessive porosity is particularly underscored. For instance, the porous structure of 3D‐printed scaffolds facilitates the delivery of nutrients to the cells, promotes cell viability, and provides the cell with a suitable media for the regeneration of organs or tissues.^21^, ^22^, ^23^ The most outstanding applications of 3D printing technology are organ fabrication, precise printing of drugs, medical phantoms, and different aspects of cancer treatment ranging from diagnosis to drug delivery.^24^, ^25^ Although 3D printing of biopolymers is well‐accepted among scientists, it generally suffers from some limitations, such as a lack of dynamism and responsiveness. Indeed, the final 3D‐printed structure fails to follow a dynamic pattern of change in shape, swelling, self‐repairing, self‐assembly, multifunctionality, and shape‐shifting properties as a function of time. On the other hand, lacking dynamism negatively affects and weakens biomimicry. Hence, 4D printing was introduced and progressed to mimic nature‐inspired structures.^26^

4D printing assists in promoting the structural configuration of printed materials as a function of time. 4D printing makes good use of biomedical, chemistry, materials, and computer science to develop advanced materials. Biomaterials with sensitivity to particular stimuli are the building blocks of 4D printing technology, which can be classified as physical, chemical, and biological stimuli‐responsive materials.^27^ Physically responsive materials are sensitive to temperature, light, humidity, electricity, and magnetic field, while chemical ones take action when a change in pH and ion concentration exists. More intricately, biological stimuli materials are responsive to cell traction forces, glucose, and enzymes.^25^, ^28^, ^29^ Similar to 3D printing, 4D printing is based on a powerful mathematical model of the desired structure/image. Likewise, 4D printing can be classified into four main technologies, including SLA, fused‐deposition modeling, powder bed, and inkjet head 3D printing. The strategy to be selected is dependent on the mechanical properties of the used biomaterials and their flexibility as well as printability.^30^

Very recently, a new class of 3D printers has been introduced, called the 5D printer. On this note, the 5D printing technique is not the next generation of 4D printing. It allows one to print curved layers by two additional axes, leading to a higher degree of freedom. This printer can move the print bed and printing head in two more angles.^31^ Besides the capability of printing complicated curved layers, 5D printers can create scaffolds possessing adequately high mechanical properties. For instance, 5D‐printed scaffold can tolerate a pressure four times, on average, higher than that tolerated in 3D‐printed scaffold. Therefore, hard and complex tissues, like bone and teeth parts can be printed accordingly.^32^ On the other hand, this class of bioprinters fails to print smart materials that reveal shape change over time. Forecasts suggest that 5D printing can support the development of surgical tools and prosthetics. Although 5D printing seems promising, there are some important blind spots and unanswered questions that need further investigations. To name but a few, the following questions still remain open:

- Does 5D printing process, itself, leave any trace on the response of the ink to the environmental clues or the seeded cells' biological functionality?
- Does the dynamics of 5D bioprinting disrupt the cells' metabolic activities seeded within the scaffold?
- Is it applicable to print scaffolds that are implantable in the body by inducing the capability of size alteration?
- Is it possible to have a 5D‐printed scaffold undergone reaction when surrounded with immune cells or under pathological circumstances?

By and large, although bioprinted scaffolds have been repetitively reported as efficient and have experienced an exemplary progression, the complexity of innate multicellular tissues jeopardizes the accuracy and biological dynamicity. Thus, and unfortunately indeed, recapitulating the real features of native tissue is the main concern.

**FIGURE 1:**
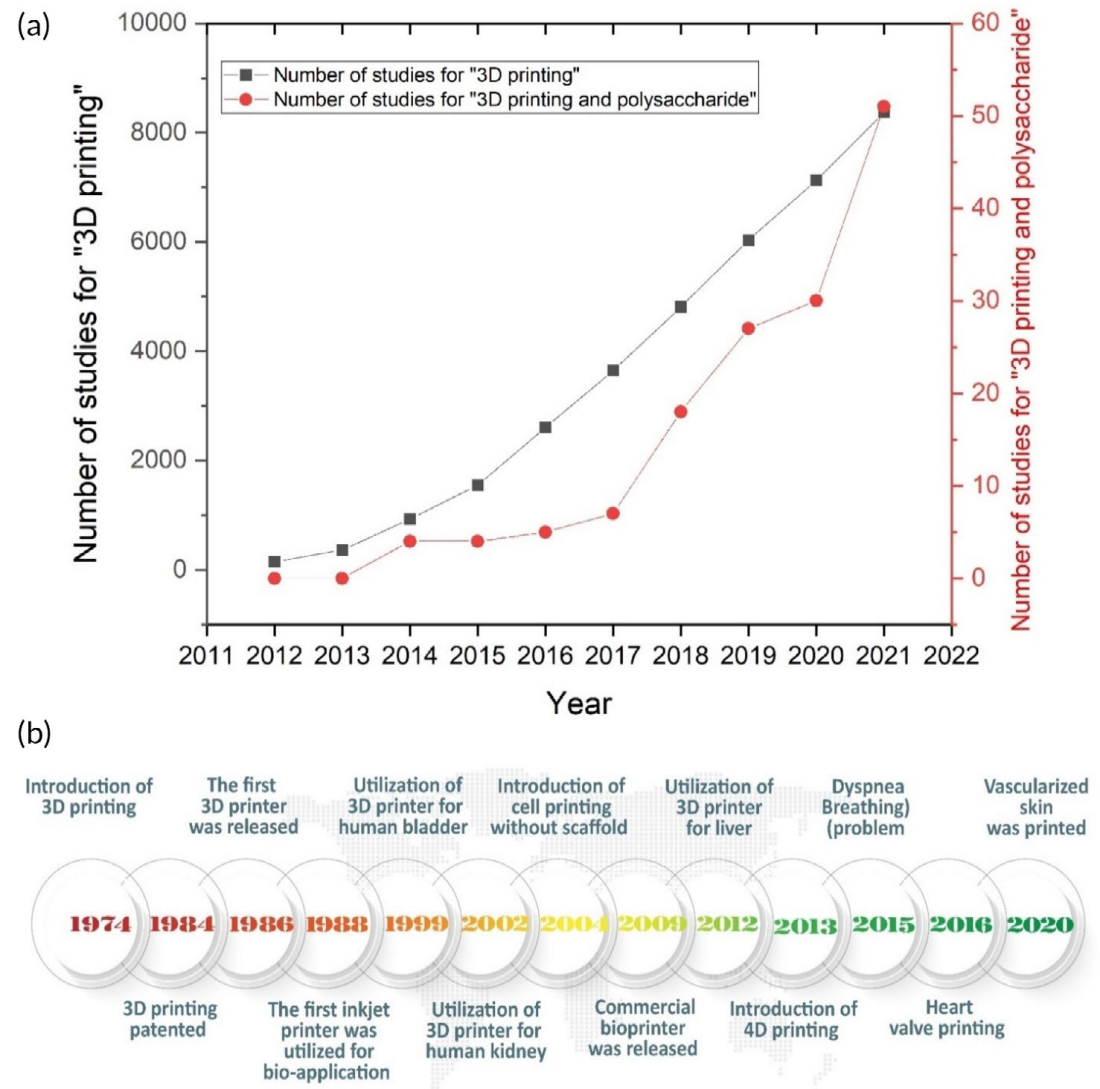
(a) Schematic illustration of the number of scientific papers published on 3D and 4D printing per year (2012–2021) and the number of papers on 3D and 4D printing of polysaccharides per year (2012–2021). (b) The history of progression from 3D to 4D printing in a short view.

Figure 1a shows the history of progression from 3D to 4D printing in the development of biomaterials for medical applications. It is evident from the timeline graph that applications of 3D and 4D printing are becoming more and more delicate, critically viable, and targeted. Moreover, attempts in using 3D and 4D printing techniques to shape polysaccharides follow an ascending trend (Figure 1b). Although progressing very fast, a long way should be paved for the appropriate selection of printable polysaccharides. Besides highly printable character, the chosen candidates should have high sensitivity to possible stimuli, great biological features, good mechanical properties, sustainability, and recyclability. In this review article, we wrote a short introduction of 3D and 4D printing concepts, followed by clarification of the need for a shift from 3D to 4D printing while considering the polysaccharides' role, and challenges associated with the application of 4D printing to polysaccharides. We have also proposed possible solutions to existing challenges.

## 2 OVERVIEW OF 3D AND 4D PRINTING TECHNOLOGIES

“3D printing is actually 2D printing over and over again,” told by Prof. J. DeSimone during a TED talk in 2017. At present, 3D printing covers a wide range of printing technologies. To name but a few, fused deposition modeling (FDM), SLA, selective laser sintering (SLS), selective laser melting (SLM), electron beam melting (EBM), inkjet 3D printing (3DP), and direct ink writing (DIW) can be addressed.^33^ All the mentioned types of 3D printers are similar in working in a layer‐by‐layer manner to print materials under a controlled computerized program. According to ISO/ASTM52900‐15, AM can also be divided into seven categories: material extrusion, vat photopolymerization, powder bed fusion, material jetting, binder jetting, sheet lamination, and directed energy deposition.^34^, ^35^ This new technology is considered invaluable due to addressing three critical concerns. First, it can provide complex geometries that are not achievable by traditional routes. Second, it can print different kinds of biopolymers simultaneously, without the need for toxic chemical reagents and solvents. Third, it leaves no waste.^36^ However, when using 3D printers, there is no opportunity to deform the scaffold. The fabricated scaffolds are nondynamic and there is a need to mimic the nature‐inspired structures using smarter materials. To resolve this situation, 4D printing is introduced utilizing advanced and smart materials showing stimuli‐responsiveness behavior. In this regard, polysaccharides received popularity in 4D printing due to their multidimensional responsiveness.^37^, ^38^ Basically, 4D printing can be classified to three main categories: liquid solidification, powder solidification, and direct material extrusion.^39^, ^40^ Shape memory polymers (SMPs), alloys (SMAs), and composites (SMCs) are smart materials suited for 4D printing.^41^ Nevertheless, not only is there more rigor in choosing materials for 4D printing, but also the expectations are very high.

In addition to the parameters mentioned in Table 1, there are some major and basic requirements for both 3D and 4D bioprinting (i) Printing parameters such as printing speed, extrusion rates, nozzle moving speed, nozzle height, and nozzle diameters, (ii) rheological parameters such as shear rate, ingredients, printing temperature, and the concentrations, and (iii) ink parameters such as biocompatibility, printability, autonomous shape memory in response to an external stimulus, viscoelasticity, in situ gelations, permeability, and biodegradation.

**TABLE 1:** Key parameters included in stimuli‐responsiveness and applications of bioprinting technologies.

Required mechanical/physical parameters of 3D‐printed scaffolds	Composition	Porosity	Stiffness	Elasticity	Predictable degradation pattern	Ease of administration	42, 43, 44
Required biological parameters of 3D‐printed scaffolds	Low immunogenicity	Mimicry to the native environment	Release of factors (if needed)	Integration with cells	Nontoxic degradation products	Biocompatibility	45, 46
How to control mechanical or biological properties							
Enzymatically	Chemically	Divalent ion concentration	Tuning pH	Tuning ionic strength	Using additives	Introducing of new moieties	47, 48, 49
Possible extra stimulus (4D)							
Temperature	PH	Ion concentration	Electric field	Light	Magnetic field	Absorption/Desorption	50, 51
Theory							
Internal stress inequality	NA	NA	Electro‐thermal effect	Photo‐thermal effect	Magnetic drive	NA	50, 52
Pros: Ease of operation Cons: Slow response	Pros: Ease of solution operation Cons: Need of pH solutions	Pros: Ease of solution operation Cons: Need of ionic solutions	Pros: Fast response Cons: Need of electrolytes and electrodes	Pros: Remotely controlled Cons: controlling over penetration of light into the depth	Pros: Remotely controlled Cons: the need for magnetic particles addition	Pros: Ease of operation Cons: Slow response	53, 54
Applications							
Neural tissue substitute	Dermal tissue engineering	Engineering of chondrocytes	Cell‐homing scaffold	Culture of Fibroblasts and Chondrocytes	Fibroblast/macrophage co‐culture	Cardiac tissue engineering	51, 55
Formulations							
Lyophilization	Photo‐cross‐linkable hydrogel	Solvent casting	Crosslinked hydrogel	Multinozzle deposition of the components	Lyophilized hydrogel	Electrospun nano‐fibers	56, 57

Abbreviation: NA, not available/applicable.

**FIGURE 2:**
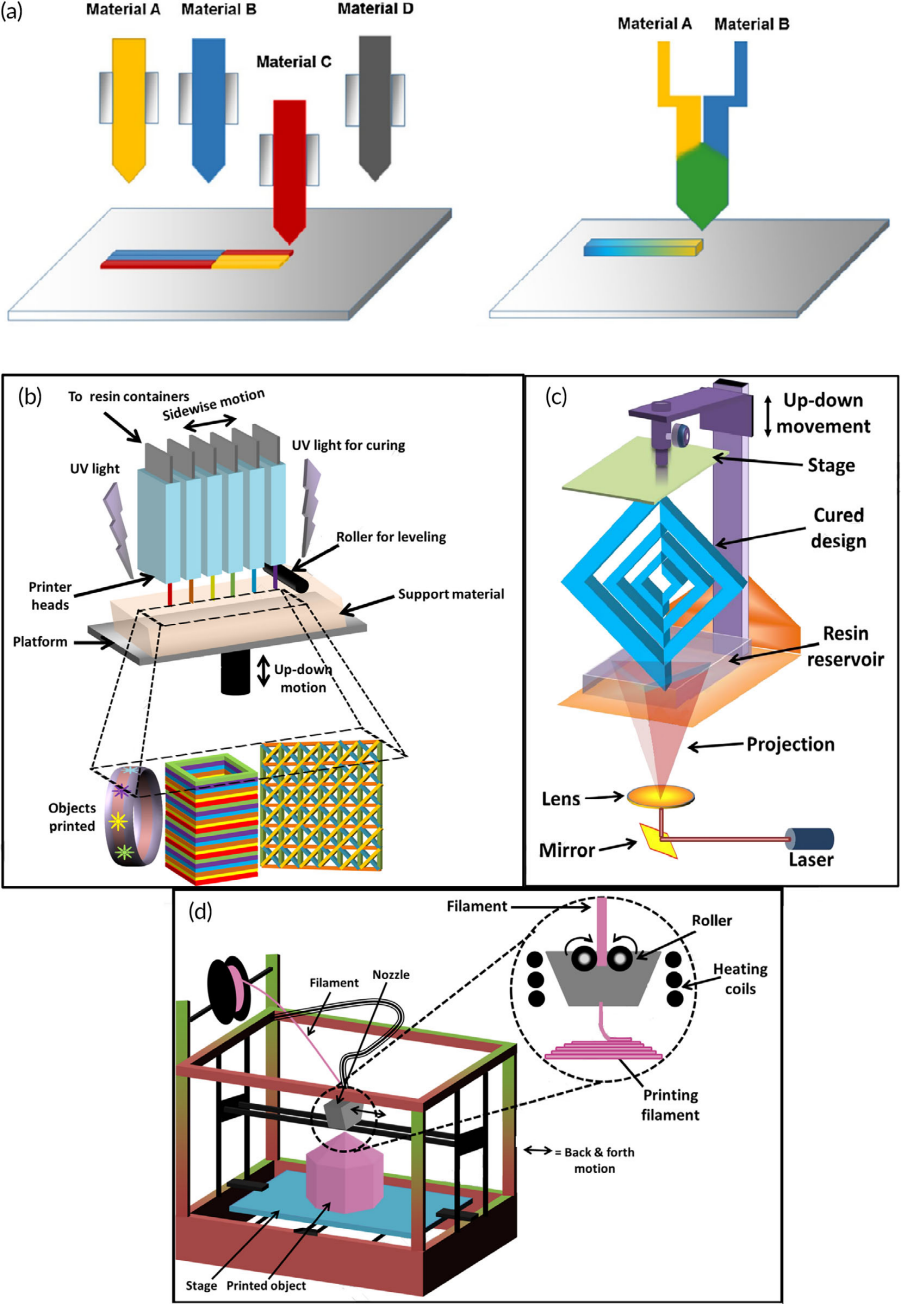
The picture reveals the schematic illustration of multimaterial printing usually utilized in 4D printing and the other common methods of printing. (a) Multimaterial extrusion method for 4D printing.^58^ (b) A schematic illustration of a multimaterials printer. (c) Illustration of the key elements in fused deposition modeling (FDM) printer. (d) Schematic of stereolithography (SLA) including a laser for curing the biomaterials, lens as a mirror and an elevator for movement.^59^

There are also some major differences between 3D and 4D: (i) materials for 4D printing are smarter, advanced, designed, or self‐assembled, while thermoplastics, metals, and ceramics are the common materials for 3D printing; (ii) 4D printing device is a multimaterial 3D printer (Figure 2)^58^; and (iii) the final scaffold achieved by 3D printing remains unchanged by the time (after applying stimuli), while in 4D printing it does change.^60^ Table 2 summarizes the comparison between 3D and 4D printing technologies.

**TABLE 2:** The comparison of 3D and 4D printing technologies in a brief view. It can be seen that printing variables and properties of the printed articles must be matched for a target application.

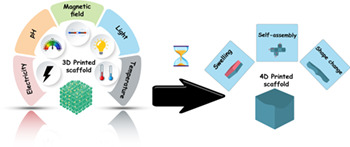				
Progression made from 3D to 4D printing technology				
Variable	3D	4D	Comments	References
Fabrication process	Layer‐by‐layer from bottom to top	Layer‐by‐layer manner (fabricating surfaces with self‐transformation ability)	There is a need for improving the resolution	61, 62
Materials	Thermoplastics, ceramics, metals, biomaterials, or nanomaterials	Multimaterial and self‐assembling materials such as polysaccharides	Multiresponsive materials are required/there is a need for privatization per application	63, 64, 65
Flexibility	Rigid	Flexible	Changing flexibility over time is needed	66, 67
Programming of material	Simple materials are mostly used	Advanced materials are mostly used	More smart and responsive materials are required	68

## 3 CHALLENGES IN BIOMATERIALS DEVELOPMENT BY 3D AND 4D PRINTING

**FIGURE 3:**
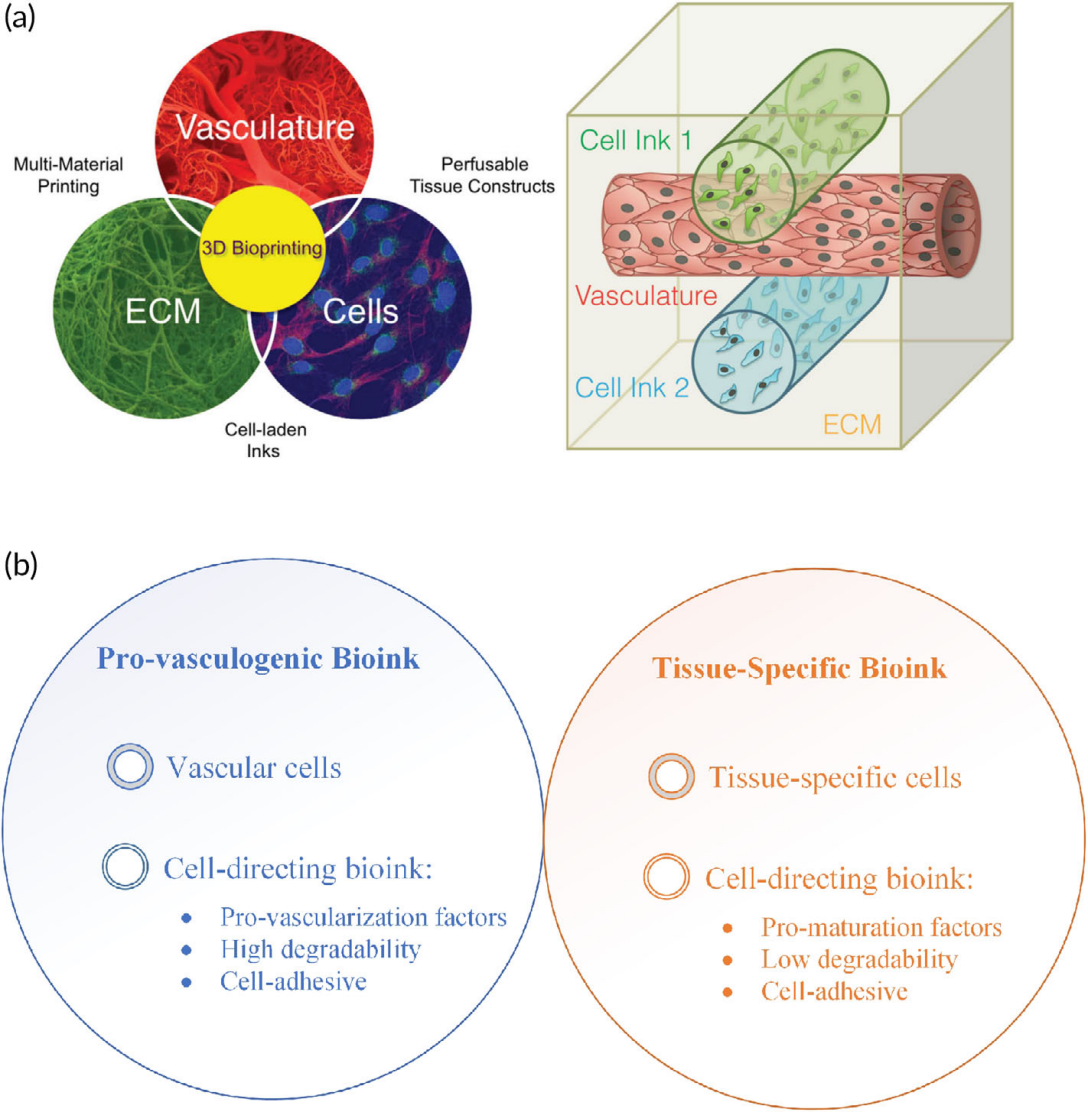
(a) Co‐printing of the vasculature, cells, and extra cellular matrix (ECM) to improve vascularization in a printed cell‐laden tissue construct.^75^ (b) A suggested 3D bioprinting strategy to fabricate vascularized tissue using the combination of 3D extrusion printing with cell‐directing materials is a multiscaled approach for printing vascularized tissue in a layer‐by‐layer manner.

There exist some challenges in the printing of biopolymers, originating from material defects. Printability, biocompatibility, biomimicry, degradation pattern, and degradation byproducts are the main limitations.^69^, ^70^, ^71^ Fortunately, there are also several possible resorts for the addressed issues. For instance, modifying commercial printers, material modifications, devising state‐of‐the‐art solvent systems, incorporation of polysaccharides with other bioactive materials, and developing some postprocessing techniques such as surface coating and plasma radiation can be counted.^72^, ^73^, ^74^ Due to the considerable biological features of polysaccharides, they can be of great interest as inks. However, their poor mechanical properties must be considered an important constraint. Additionally, manipulation of multiple biomaterials and cell types is necessary to actualize the printing of a vascularized and metabolically active thick tissue, which is technically called biomimicry (Figure 3).^76^, ^77^, ^78^

4D printing of biomaterials enables the manufacturing of complex architectures and composition of natural tissues such as the heart and kidney. The dynamic nature of 4D printing technology provides the user with an opportunity to increase the biomimicry of the final scaffold.^79^ To date, evaluations of 4D printing of natural biomaterial has been successfully guided in different forms such as beads, channels, rolls as well as sheets. This diversity combined with the dynamic basis of 4D printing could potentially enhance biomimicry. However, most of the printed tissues are suffering from being avascular, aneural, and lymphatic.^80^ Remarkably, the correct utilization of biomaterials plays a key role in improving the angiogenesis effect.^81^ Hence, not only correct decision‐making about the fabrication method (4D bioprinting) but also opting for smart biomaterials will leave an essential trace on vascularization.^81^ Additionally, there are some unanswered questions to be addressed. For instance, what are the possible effects of material responses on cell metabolic activities? Does material dynamics affect the cell viability? What is the effect of the cell seeding on the material responsiveness? How responsive materials react when surrounded with immune system?

**FIGURE 4:**
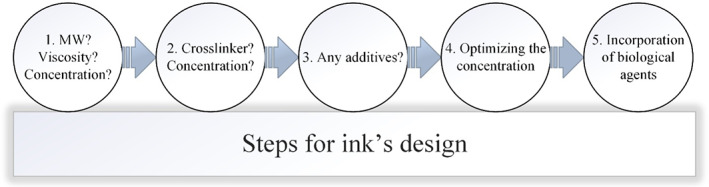
The picture reveals the schematic illustration of the necessary steps to successfully design bioinks for bioprinting. Accordingly, we need to designate the molecular weight (MW), viscosity, and concentration of the used biomaterials based on the application (Step 1). Then, the crosslinker and its concentration must be defined in order to improve the properties of the bioink (Step 2). The additives are usually added in an optimized manner in order to add a special characteristic to the final printed scaffold (Step 3). Then, standard tests will be performed to determine the optimized concentration in which the platform has the best properties (Step 4). Finally, biological agents (e.g., drug, growth factors, macromolecules) would be incorporated, if needed.

Although polysaccharides are one of the main existing options for designing printing inks (Table 3 and Figure 4), some reports have indicated that their inappropriate shape‐morphing ability is a serious limitation associated with the 4D printing of polysaccharides. However, other excellent properties of these biomaterials such as biocompatibility, nontoxicity, and abundance cannot be ignored. Hence, scientists have suggested overcoming their shape‐morphing issues by blending with other biopolymers.^103^ For instance, alginate's undesired shape‐morphing ability can be resolved when it is mixed with methylcellulose or dopamine. The resulting hydrogel has great rheological properties, shape‐morphing ability, and extrudability.^104^, ^105^ Another example of improving shape morphing capability of polysaccharides via blending is the addition of multiwalled carbon nanotubes, which brings not only an efficient photothermal conversion capability (a photo‐responsive shape‐changing composite) but also stronger mechanical properties.^106^ However, sometimes the additives are cytotoxic and we need to tradeoff between the biocompatibility of the additive and shape‐morphing capability of the resultant composite. For instance, high concentrations of carbon nanotubes induce cell apoptosis necessitates the design of safer additives.^107^

**TABLE 3:** Different steps of designing polysaccharide‐based inks/applications of polysaccharides and polysaccharide‐based inks in 3D and 4D printing, their benefits and their challenges.

Different inks' printing parameters	Viscosity	Concentration of the effective material	Transparency	Density	Thermal resistance	Printability	82, 83, 84
Inks	Alginate	Agarose	Cellulose	Methyl cellulose	Gum	Hyaluronic acid	22, 85
Applications	Adipose tissue, bone tissue, chondrocyte and cartilage tissue, fibroblast and vascular constructs, hepatocytes, mesenchymal stem cells as well as neural tissue	Chondrocyte and cartilage tissue, endothelial cells, mesenchymal stem cells, neural stem cells as well as neural tissue	Adipose tissue‐derived stem cells, chondrocyte and cartilage tissue, mesenchymal stem cells as well as pluripotent stem cells	Chondrocyte and cartilage tissue, mesenchymal stem cells, pancreatic cells as well as plant cells	Chondrocyte and cartilage tissue, mesenchymal stem cells, bone tissue	Chondrocyte and cartilage tissue, neural tissue, and Schwann cells	86, 87, 88
Printing	Ink						
3ِD	Cellulose/hemi			Starch	Alginate/agarose	Chitosan	22, 89, 90
3ِD	Ink form						
3ِD	Suspension/solution hydrogel filament			Solution hydrogel filament	Solution hydrogel filament	Solution hydrogel filament	91, 92
3ِD	Challenges						
3ِD	Optimization of biodegradation and neo‐tissue formation			Optimization of mechanical and biological properties	Printing of fully and dense vascularized organs	Printing of metabolically active organs	93, 94
3ِD	Large‐scale bioprinting			High cost	In situ printing of cells	Limited printable options (materials)	
3ِD	Optimization of printing speed and the output resolution			Long duration of printed objects			
4D	Ink						
4D	Hyaluronic acid			Chitosan	Alginate	Cellulose	95, 96, 97
4D	Better control over						
4D	Biomimetic ECM			Anatomical shape	Porous structure	Real‐time cell behavior	98, 99
4D	Benefits						
4D	Simplicity of fabrication			Free from postprocessing	Spatiotemporally controllable	Optimized performances	37, 100
4D	Shape transformation after implant			Improved patient compliance	Better adaptability	Graded microarchitectures consistent with natural organ	
4D	Challenges						
4D	Optimization of cell concentration for cell‐laden scaffolds is needed			Stimuli diverse materials are needed	Stronger sensitivity and longer durability are needed	More resolution for microstructures is required	101, 102
4D	Higher printing efficacy is needed			Interdisciplinary techniques such as machine learning need to be added	A holistic understanding of regenerative medicine needs to be done to be incorporated into the printing process	Cross the gap from cell to animal model	

## 4 RATIONALE BEHIND SHIFT FROM 3D TO 4D PRINTING TECHNOLOGY

Researchers believe that 4D printing technology will cause a huge evolution in all fields, especially medicine. This new technology will improve the quality of life. For instance, today, there is an urgent need for implantable medical devices that can grow as per the patient's growth. 4D printing is capable to meet this requirement because the printed scaffolds can change their shape and structure as the organ grows. In this regard, with the help of scanning technologies such as computed tomography (CT) and magnetic resonance imaging (MRI), the growth pattern of each patient would be captured and the shape configuration of the 4D‐printed scaffold could be tuned.^108^, ^109^, ^110^, ^111^, ^112^ This technology is also able to innovate new routes for more advanced research,^98^ helpful for analyzing body defects and regeneration,^113^ and deep scanning of organs to know whether they can perform their required function,^114^, ^115^ fabricating complex medical devices as per each patient's anatomy, implementation of complex printed models in heavy surgeries in which human intervention is either difficult or dangerous as well as fabrication of internal structures with a high level of flexibility.^116^

## 5 POLYSACCHARIDES ADAPTED TO 3D AND 4D PRINTING

**FIGURE 5:**
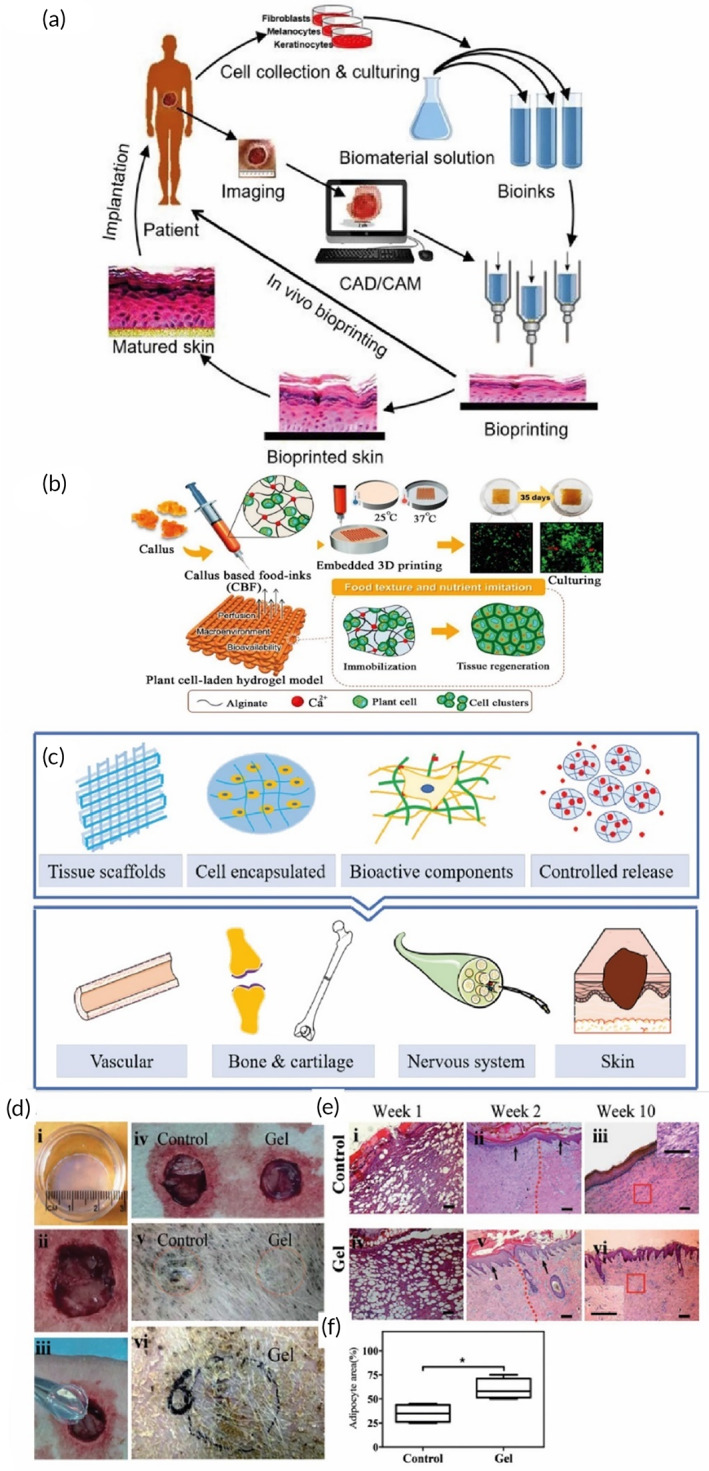
(a) The basic process depicting the 3D printing of polysaccharides‐based skin scaffold under the sufficient condition to achieve implantable mature skin.^86^ (b) Utilization of alginate‐based 3D‐printed scaffold for plant cell culturing.^121^ (c) Different applications of bioprinted polysaccharides in tissue engineering. (d) Illustration of surgical procedure for implanting the printed scaffold. (e) Histological assessment of wound healing process after 1, 2, and 10 weeks. (f) Quantitative diagram of regenerated adipocyte area 1 week after the implantation.^85^, ^122^

Natural biopolymers such as polysaccharides and proteins are of great interest in bioprinting technology. However, they should be printable in nature. Their great biocompatibility, availability, low environmentally impactful, biodegradability, low cost, nontoxicity, facile modification because of accessible functional groups, cytocompatibility, stimuli responsiveness, gelation behavior, antimicrobial activity, as well as their ability to form hydrogel have made them the best choice among biomaterials for 3D and 4D bioprinting.^117^ In the form of a hydrogel, they can be easily utilized in pressure‐assisted micro‐syringe and inkjet techniques, such that the final scaffold reveals high porosity and interconnectivity, particularly the ability to cell culture and drug loading^118^, ^119^, ^120^ (Figure 5).^121^

### 5.1 Polysaccharides in 3D bioprinting

Although several polysaccharides have been examined for printability potential, only a few of them reveal thermal stability in terms of melt strength or viscosity in printing. The overall strategy is to blend them together in order to have a complementary character, for example, mechanically stable printable polysaccharides. Table 4 shows the printing type and application of several types of bioprinted polysaccharide‐based scaffolds in biomedical engineering. It is apparent from the table that alginate is the key element among printable polysaccharides, individually or in the form of a blend with other polysaccharides and/or nanoparticles.

**TABLE 4:** Printing type and application of the printed polysaccharide‐based scaffolds. Alginate is of majority in printable formulations.

Materials	Printing type	Response to stimuli/biomedical application	References
Agarose/acrylamide	Situ polymerizing	Temperature/human ear or nose printing	123
Agarose/alginate‐aniline tetramer hydrogel	Not available (promising for further studies)	Voltage/nerve graft	124
Alginate glycerin hydrogel	Microfluidic coaxial extrusion	PH/skin dressing	125
Chitosan	Plasma polymerization	PH/surface modification	126
Chitosan/methacrylated alginate	Extrusion bioprinter	Voltage/vascular stents	127
Chitosan and native starch	Not available (promising for further studies)	Enzyme/orthopedic implant	128
Hyaluronic acid/polycaprolactone	Laser sinter	Tension/tracheobronchial splint	129
Hyaluronic acid/polylactide	Fused deposition modeling	Temperature/orthopedic implant	130
Sodium alginate/agarose/*N*, *N*′‐methylenebis (acrylamide)	Laser‐machining and screen printing	Temperature/patch	131
Alginate	Extrusion‐based printing	‐/regenerate the jaw bone	132
Alginate	Extrusion‐based printing	‐/tissue scaffolds	133
Alginate/gelatin, methacryloyl	Extrusion‐based printing	‐/hydrogel fibers	134
Alginate/graphene oxide	Micro‐extrusion process	‐/cartilage tissue engineering	135
Alginate/hyaluronic acid	Extrusion‐based printing	‐/tissue scaffolds	136
Alginate/poly(ethylene glycol)/Satureja cuneifolia plant extract	Extrusion‐based printing	‐/anti‐diabetic	137
Alginate/ poly(ε‐caprolactone)	Extrusion‐based printing	‐/auricle regeneration	138
Alginate/polyacrylate	Extrusion‐based bioprinting	‐/skin sensor	139
Alginate/agar	Thermal‐assisted 3D printing	‐/tissue scaffolds	140
Alginate/cellulose	Extrusion‐based printing	‐/human lipoaspirate‐derived adipose tissue	141
Alginate/gelatin	Extrusion‐based printing	‐/tissue scaffolds	142
Alginate/gelatin	Extrusion‐based printing	‐/nerve scaffolds	143
Alginate/gelatin/cellulose	Extrusion‐based printing	‐/tissue scaffolds	144
Alginate/hyaluronic acid	Extrusion‐based printing	‐/tissue scaffolds	145
Alginate/methylcellulose	Extrusion‐based bioprinting	‐/bone tissue engineering	146
Alginate/methylcellulose	Extrusion‐based printing	‐/wound healing	147
Alginate/methylcellulose	Extrusion‐based printing	‐/complex‐shaped tissue constructs	148
Alginate/poly(vinyl alcohol)/silk fibroin	Extrusion‐based printing	‐/maxillofacial surgery	149
Bioactive glasses and alginate	Extrusion‐based printing	‐/hard tissue application	150
Cellulose nanofibril/alginate/lignin	extruding and shaking technique	‐/cell culture	151
Cellulose, alginate	Extrusion‐based printing	‐/imminent antimicrobial	152
Gelatin/alginate/nano‐silicate	Extrusion‐based printing	‐/Bone healing	153
Hyaluronic acid/alginate	Extrusion‐based printing	‐/cartilage engineering	154
Oxidized‐alginate/micro‐gelatin particles	Extrusion‐based printing	‐/complex‐shaped tissue constructs	155
Polydopamine/alginate gelatin	Extrusion‐based printing	‐/tissue scaffolds	156

**FIGURE 6:**
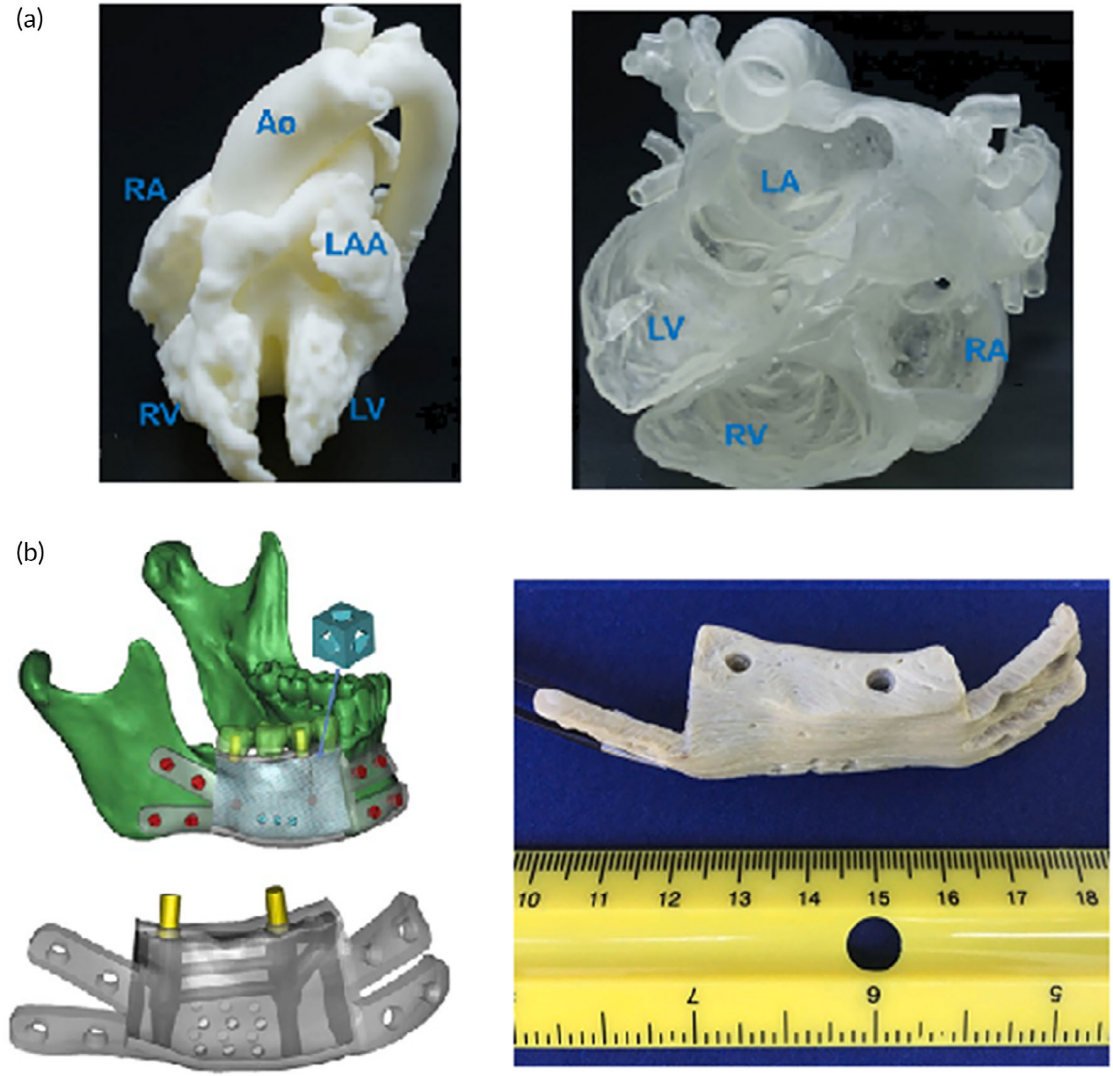
A schematic illustration of (a) 3D‐printed heart model reported by several papers in literature,^162^, ^166^ (b) 3D‐printed bone model, both the computer design before printing and the actual printed model.^167^

Polysaccharide‐based 3D printed scaffolds can support the homogeneous distribution of functional chondrocytes in addition to the retention of chondrocyte phenotype.^157^ Hence, they seem to be a potent option for clinical uses. The possibility of nanofibers fabrication from cellulose acetate and chitosan can endorse exploiting them for regulation of morphology and tuning the release profile of the printed scaffold.^158^, ^159^ Of particular note, chitosan is well known in the biomedical engineering and bioprinting industry due to its great ability to mimic the heart, bone, cartilage, vascular, skin, and neuronal extracellular matrix^160^, ^161^, ^162^, ^163^, ^164^, ^165^ (see Figure 6, ^166^, ^167^). It also enjoys repairability due to its ability to cell attachment and cell differentiation. However, its mechanical properties and printability pose a limitation on its usage in digital printing. In addition, printing accuracy and resolution of the ultimate bioprinted scaffold must be carefully supervised.

Thus, chitosan should be modified with other polysaccharides to resolve its poor printability. The addition of polyethylene glycol (PEG), gelatin, and pectin can guarantee the facile extrusion of chitosan by controlling its viscosity.^168^, ^169^, ^170^, ^171^ From this perspective, some believe that chitosan is a modifier rather than a continuous phase in the formulation of polysaccharide‐based biomaterials for 3D printing. Moreover, the presence of imine bonds between oxidized hyaluronate and glycol chitosan as well as the acyl hydrazone bonds between oxidized hyaluronate and adipic acid, the dihydrazide can result in the development of a highly printable chitosan‐based platform with self‐healing capability.^172^, ^173^ Neat polysaccharides, particularly cellulose and lignin, severely boost the mechanical strength of chitosan inks in comparison to proteins such as gelatin.^174^, ^175^ Unlike chitosan with limited printability potential, alginate has attracted a great deal of attention because of its excellent printability. Moreover, biocompatibility, low cost, low toxicity, and fast gelation (when Ca^2+^ exists as a cross‐linker^176^, ^177^) are other characteristics of alginate. This is the reason for the diversity of investigations carried out to print alginate‐based inks and their rapid bioprinting progression.^121^, ^178^ However, some reported low viscosity of alginate‐based inks. The low viscosity of alginate‐based inks can be compensated for by combination with chitosan, poly(vinyl alcohol), or hydroxyapatite.^179^, ^180^ Nguyen et al. claimed that a combination of alginate and cellulose in 3D printing supports pluripotent stem cell growth. The suggested platform can hopefully be utilized for cartilage tissue engineering.^159^, ^181^ Additionally, 3D‐printed collagen–alginate scaffolds are useful for chondrocyte culturing.^182^, ^183^ Interestingly, a recent research has served alginate as an excellent option for ultrafast 5D printing. They demonstrated that the resulting scaffold was extremely porous, with high similarity and great bio‐interaction and integration with the native tissue.^184^, ^185^

Besides alginate, chitosan, and their blends, some other polysaccharides are occasionally applied in 3D printing. Pectin provides the user with a great media for cell attachment, and cell organization as well as primary human cells, mesenchymal stem cells, fibroblasts, and osteoblasts growth. However, weak shear‐thinning properties can limit its practicality for 3D printing. The addition of other biopolymers to pectin was accordingly examined. The incorporation of carboxylated cellulose nanofibrils into pectin not only enhanced its viscoelastic behaviors but also its printability and shear‐thinning properties.^186^, ^187^ Similarly, methylcellulose can intelligently be utilized to strengthen the bonds among the printed layers of alginate‐based inks. Li et al. demonstrated that the presence of methylcellulose and trisodium citrate as a chelating agent within an alginate ink not only increases the thixotropic features but also the extrudability.^188^, ^189^ Besides, pectin can form polyelectrolytes via physical crosslinking of its carboxylic groups with the amino groups of chitosan in some specific ranges of PH (between 3 and 6). Hence, the combination of pectin with chitosan leads to a modification of the printability of chitosan.^168^, ^169^, ^170^, ^171^ Moreover, the introduction of photo‐crosslinkable methacrylic units to the polysaccharides' backbone, for example, pullulan, positively affects its printability. Functionalization of pullulan with extracellular matrix proteins can also bring about appropriate cell adhesion, especially adhesion to mesenchymal and epithelial cells.^190^, ^191^, ^192^

### 5.2 Polysaccharides in 4D bioprinting

**FIGURE 7:**
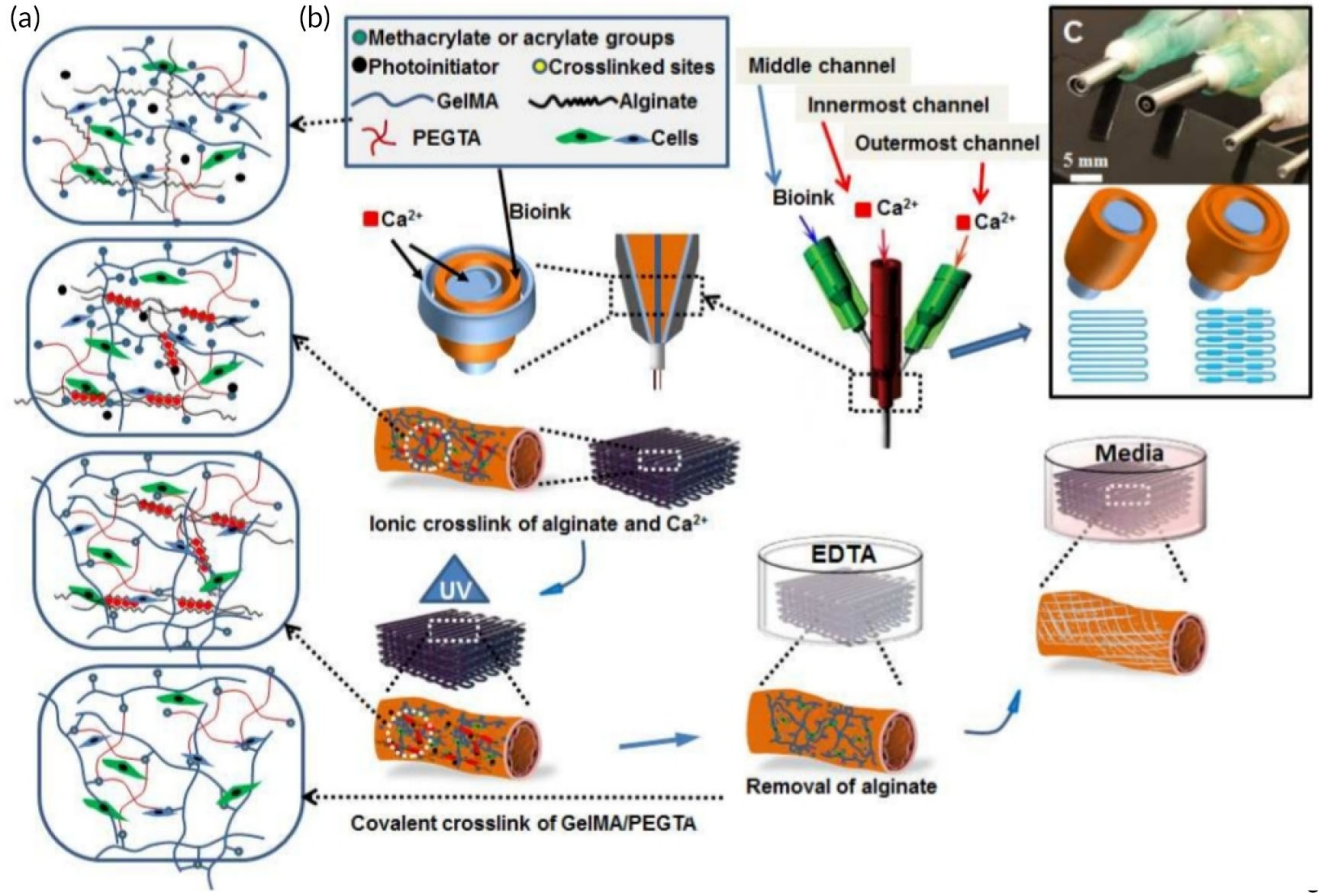
(a) The Schematic illustration of the chemical crosslinking of alginate with PEG via exposing them to CaCl_2_ solution and UV light. The presence of PEG activates temperature and salt concentration responsiveness which is a key factor in 4D bioprinting, (b) Crosslinking mechanism after material being surrounded by ca^2+^ ions and exposure to UV light, (c) Diameter of the 4D bioprinter and the pattern with which the target scaffold is printed.^200^

To be used in 4D printing, materials must own sensitiveness to a particular stimulus (or multistimuli), as mentioned earlier. These stimuli can be chemical, physical, or even biological. However, they have to provide shape change as a function of time, after applying the motives. The stimuli responsiveness of polysaccharides will provide us with the opportunity to utilize them in 4D printing technology. They can easily respond to physical stimuli like temperature, light, electricity, magnetic field, or even pressure, chemical species such as reactive oxygen species (ROS), redox species (e.g., glutathione), glucose, enzymes, and some ions (e.g., calcium).^193^, ^194^ For example, chitosan is responsive to glucose,^195^ pH (under acidic conditions, due to the presence of basic amine groups),^127^ or even an electric field.^196^ Moreover, reports have indicated that agarose, sodium alginate, and hyaluronic acid respond to temperature deviation, chitosan and agarose react to voltage changes, alginate glycerin arouses in response to PH, and hyaluronic acid is affected when tension is applied^197^ (see Table 4). Additionally, a combination of cellulose, dextran, and graphene reveals pH and near‐infrared (NIR) sensitive properties.^198^ Noteworthily, some of them have multiresponsiveness to more than one stimulus.^199^ There are methods to modify polysaccharides preparing them as 4D bioprinting's inks. The introduction of hydrophobic, acidic, basic, or other chemical functional groups on their backbone makes changes in some of their properties such as stimuli responsiveness. The main chemical reactions that have been used more in this regard are enzymatic reactions, oxidation reactions, or nucleophilic reactions (see Figure 7).^201^, ^202^

## 6 CONCLUDING REMARKS AND FUTURE PERSPECTIVE

In this manuscript, we reviewed the concepts of 3D and 4D bioprinting technologies, their limitations, and the role of polysaccharides in the development of bioprinting. We also presented a short introduction to the 5D printing advent. Although plenty of research has focused on the reduction of 3D printing costs and increasing its quality, slow speed of printing and expensiveness still appear as the main drawbacks. It is worth mentioning that the method proposed for ultrafast printing suggested by Huang et al. seems promising but needs further investigations to actualize the scale‐up process.^203^ One possible way to increase the printing speed is the introduction of supramolecular interactions or self‐assembled structures.^48^, ^204^ Moreover, since printing accuracy can leave an essential trace on the outcome quality of the experiments, researchers must be cautious about the quality and printability of the inks. When it comes to the printability of some polysaccharides, one can utilize different methods such as blending with other biopolymers, dispersing some additives to increase the printability as well as using chemical crosslinking strategies.^205^ A holistic understanding of the required printing factors is essential to overcome the barriers related to printability, precision, and accuracy.^206^ Additionally, there exist some other challenges related to both 3D and 4D printing of polysaccharides. For instance, intemperate interconnectivity, thick structure as well as very low viscosity are the problems that some polysaccharide‐based inks are suffering from.^92^, ^207^

There exist two possible clarifications in this direction. First, advanced material design has to be contemplated to improve printability, mechanical properties, and biological features. Second, the advanced digital simulation needs to be mature and enhanced, leading to the fabrication of smarter materials. Although plenty of efforts should be integrated into a protocol to resolve 4D printing challenges, it is believed that 4D printing technology would find amazing applications in the near future. For instance, it would become a unique method of surgery to implant medical devices more efficiently. Using the state‐of‐the‐art 4D printing technique, we would be able to provide the surgeon with all the needed data about blood loss, blood clots, as well as breathing difficulties. Moreover, smart devices could prepare detailed information about the anatomies of the individual patient (at anytime and anywhere after the surgery), as an impossible task in the past.^98^ Considerably, we believe that 5D printing will also have a bright future, especially in cancer treatment. The ability to monitor the distortion of the tumor's anatomy, the possibility of tumor invasion to the surrounding structure, and monitoring the possible changes occurring after neoadjuvant treatments are the important factors that would help complicated surgical planning using 5D bioprinters, an interesting subject that has been recently studied by a group of scientists.^208^ What we may need to take huger steps is a deeper understanding of the interaction of the printed organ with the host tissue and the native microenvironment, the possible response of the printed organ to the body's immune system, and the pathological conditions.^209^ By far, many studies have to be conducted and plenty of challenges must be resolved to reach such a stage of bioprinting knowledge.

## AUTHOR CONTRIBUTIONS

**Hanieh Shokrani:** Formal analysis (equal); validation (equal); writing – original draft (lead). **Amirhossein Shokrani:** Formal analysis (equal); graphics (equal); writing – original draft (equal). **Farzad Seidi:** Data curation (equal); validation (equal); Supervision (equal). **Mohammad Mashayekhi:** Graphics (equal); writing – original draft (supporting). **Saptarshi Kar:** Data curation (equal); methodology (supporting). **Dragutin Nedeljkovic:** Formal analysis (equal). **Tairong Kuang:** Formal analysis (equal); visualization (equal); writing – original draft (supporting). **Mohammad Reza Saeb:** Supervision (equal); methodology (equal); visualization (lead); validation (supporting); writing – review and editing (lead). **Masoud Mozafari:** Investigation (equal); methodology (equal); supervision (equal); validation (supporting); writing – review and editing (lead).

## CONFLICT OF INTEREST STATEMENT

The authors have no conflict of interest to declare.

### PEER REVIEW

The peer review history for this article is available at https://publons.com/publon/10.1002/btm2.10503.

## Data Availability

The authors confirm that the data supporting the findings of this study are available within the article.
